# The met and unmet health needs for HIV, hypertension, and diabetes in rural KwaZulu-Natal, South Africa: analysis of a cross-sectional multimorbidity survey

**DOI:** 10.1016/S2214-109X(23)00239-5

**Published:** 2023-08-15

**Authors:** Urisha Singh, Stephen Olivier, Diego Cuadros, Alison Castle, Yumna Moosa, Thando Zulu, Jonathan Alex Edwards, Hae-Young Kim, Resign Gunda, Olivier Koole, Ashmika Surujdeen, Dickman Gareta, Day Munatsi, Tshwaraganang H Modise, Jaco Dreyer, Siyabonga Nxumalo, Theresa K Smit, Greg Ordering-Jespersen, Innocentia B Mpofana, Khadija Khan, Zinzile E L Sikhosana, Sashen Moodley, Yen-Ju Shen, Thandeka Khoza, Ngcebo Mhlongo, Sanah Bucibo, Kennedy Nyamande, Kathy J Baisley, Alison D Grant, Kobus Herbst, Janet Seeley, Deenan Pillay, Willem Hanekom, Thumbi Ndung’u, Mark J Siedner, Frank Tanser, Emily B Wong, Vukuzazi team, Emily B. Wong, Stephen Olivier, Resign Gunda, Olivier Koole, Ashmika Surujdeen, Dickman Gareta, Day Munatsi, Tswaraganang H. Modise, Jaco Dreyer, Siyabonga Nxumalo, Theresa K. Smit, Greg Ording-Jespersen, Innocentia B. Mpofana, Khadija Khan, Zizile E.L. Sikhosana, Sashen Moodley, Yen-Ju Shen, Thandeka Khoza, Ngcebo Mhlongo, Sana Bucibo, Kennedy Nyamande, Kathy J. Baisley, Diego Cuadros, Frank Tanser, Alison D. Grant, Kobus Herbst, Janet Seeley, Willem A. Hanekom, Thumbi Ndung'u, Mark J. Siedner, Deenan Pillay, Mosa Suleman, Jaikrishna Kalideen, Ramesh Jackpersad, Kgaugelo Moropane, Boitsholo Mfolo, Khabonina Malomane, Hlolisile Khumalo, Nompilo Buthelezi, Nozipho Mbonambi, Hloniphile Ngubane, Thokozani Simelane, Khanyisani Buthelezi, Sphiwe Ntuli, Nombuyiselo Zondi, Siboniso Nene, Bongumenzi Ndlovu, Talente Ntimbane, Mbali Mbuyisa, Xolani Mkhize, Melusi Sibiya, Ntombiyenkosi Ntombela, Mandisi Dlamini, Hlobisile Chonco, Hlengiwe Dlamini, Doctar Mlambo, Nonhlahla Mzimela, Zinhle Buthelezi, Zinhle Mthembu, Thokozani Bhengu, Sandile Mtehmbu, Phumelele Mthethwa, Zamashandu Mbatha, Welcome Petros Mthembu, Anele Mkhwanazi, Mandlakayise Sikhali, Phakamani Mkhwanazi, Ntombiyenhlahla Mkhwanazi, Rose Myeni, Fezeka Mfeka, Hlobisile Gumede, Nonceba Mfeka, Ayanda Zungu, Hlobisile Gumede, Nonhlahla Mfekayi, Smangaliso Zulu, Mzamo Buthelezi, Senzeni Mkhwanazi, Mlungisi Dube, Philippa Matthews, Siphephelo Dlamini, Hosea Kambonde, Lindani Mthembu, Seneme Mchunu, Sibahle Gumbi, Tumi Madolo, Thengokwakhe Nkosi, Sibusiso Mkhwanazi, Simbusio Nsibande, Mpumelelo Steto, Sibusiso Mhlongo, Velile Vellem, Pfarelo Tshivase, Jabu Kwinda, Bongani Magwaza, Siyabonga Nsibande, Skhumbuzo Mthombeni, Sphiwe Clement Mthembu, Antony Rapulana, Jade Cousins, Thabile Zondi, Nagavelli Padayachi, Freddy Mabetlela, Simphiwe Ntshangase, Nomfundo Luthuli, Sithembile Ngcobo, Kayleen Brien, Sizwe Ndlela, Nomfundo Ngema, Nokukhanya Ntshakala, Anupa Singh, Rochelle Singh, Logan Pillay, Kandaseelan Chetty, Asthentha Govender, Pamela Ramkalawon, Nondumiso Mabaso, Kimeshree Perumal, Senamile Makhari, Nondumiso Khuluse, Nondumiso Zitha, Hlengiwe Khati, Mbuti Mofokeng, Nomathamsanqa Majozi, Nceba Gqaleni, Hannah Keal, Phumla Ngcobo, Costa Criticos, Raynold Zondo, Dilip Kalyan, Clive Mavimbela, Anand Ramnanan, Sashin Harilall

**Affiliations:** a Africa Health Research Institute, KwaZulu-Natal, South Africa; b Nelson R Mandela School of Medicine, University of KwaZulu-Natal, Durban, South Africa; c School of Nursing and Public Health, University of KwaZulu-Natal, Durban, South Africa; d School of Clinical Medicine, University of KwaZulu-Natal, Durban, South Africa; e College of Health Sciences, and Centre for the AIDS Programme of Research in South Africa, University of KwaZulu-Natal, Durban, South Africa; f Digital Epidemiology Laboratory, Digital Futures, University of Cincinnati, Cincinnati, OH, USA; g Division of Infectious Diseases, Massachusetts General Hospital, Boston, MA, USA; h Ragon Institute, Massachusetts General Hospital, Boston, MA, USA; i Harvard Medical School, Harvard University, Boston, MA, USA; j International Institute for Rural Health, University of Lincoln, Lincoln, UK; k Department of Biostatistics and Bioinformatics, Rollins School of Public Health and Department of Biomedical Informatics, Emory University School of Medicine, Emory University, Atlanta, GA, USA; l Department of Population Health, New York University Grossman School of Medicine, New York University, New York, NY, USA; m London School of Hygiene and Tropical Medicine, London, UK; n Department of Pulmonology and Critical Care, Inkosi Albert Luthuli Hospital, Durban, South Africa; o School of Public Health, University of Witwatersrand, Johannesburg, South Africa; p Department of Science and Innovation, Medical Research Council, South African Population Research Infrastructure, Durban, South Africa; q Division of Infection and Immunity, University College London, London, UK; r HIV Pathogenesis Programme, Doris Duke Medical Research Institute, Durban, South Africa; s School of Data Science and Computational Thinking, Stellenbosch University, Stellenbosch, South Africa; t Division of Infectious Diseases, University of Alabama at Birmingham, Birmingham, AL, USA

## Abstract

**Background:**

The convergence of infectious diseases and non-communicable diseases in South Africa is challenging to health systems. In this analysis, we assessed the multimorbidity health needs of individuals and communities in rural KwaZulu-Natal and established a framework to quantify met and unmet health needs for individuals living with infectious and non-communicable diseases.

**Methods:**

We analysed data collected between May 25, 2018, and March 13, 2020, from participants of a large, community-based, cross-sectional multimorbidity survey (Vukuzazi) that offered community-based HIV, hypertension, and diabetes screening to all residents aged 15 years or older in a surveillance area in the uMkhanyakude district in KwaZulu-Natal, South Africa. Data from the Vukuzazi survey were linked with data from demographic and health surveillance surveys with a unique identifier common to both studies. Questionnaires were used to assess the diagnosed health conditions, treatment history, general health, and sociodemographic characteristics of an individual. For each condition (ie, HIV, hypertension, and diabetes), individuals were defined as having no health needs (absence of condition), met health needs (condition that is well controlled), or one or more unmet health needs (including diagnosis, engagement in care, or treatment optimisation). We analysed met and unmet health needs for individual and combined conditions and investigated their geospatial distribution.

**Findings:**

Of 18 041 participants who completed the survey (12 229 [67·8%] were female and 5812 [32·2%] were male), 9898 (54·9%) had at least one of the three chronic diseases measured. 4942 (49·9%) of these 9898 individuals had at least one unmet health need (1802 [18·2%] of 9898 needed treatment optimisation, 1282 [13·0%] needed engagement in care, and 1858 [18·8%] needed a diagnosis). Unmet health needs varied by disease; 1617 (93·1%) of 1737 people who screened positive for diabetes, 2681 (58·2%) of 4603 people who screened positive for hypertension, and 1321 (21·7%) of 6096 people who screened positive for HIV had unmet health needs. Geospatially, met health needs for HIV were widely distributed and unmet health needs for all three conditions had specific sites of concentration; all three conditions had an overlapping geographical pattern for the need for diagnosis.

**Interpretation:**

Although people living with HIV predominantly have a well controlled condition, there is a high burden of unmet health needs for people living with hypertension and diabetes. In South Africa, adapting current, widely available HIV care services to integrate non-communicable disease care is of high priority.

**Funding:**

Fogarty International Center and the National Institutes of Health, the Bill & Melinda Gates Foundation, the South African Department of Science and Innovation, the South African Medical Research Council, the South African Population Research Infrastructure Network, and the Wellcome Trust.

**Translation:**

For the isiZulu translation of the abstract see Supplementary Materials section.

## Introduction

Infectious diseases, including HIV and tuberculosis, have dominated the burden of disease in sub-Saharan Africa for decades.^1^ However, similar to other low-income and middle-income countries, regions within sub-Saharan Africa are experiencing an epidemiological transition in which the prevalence of chronic non-communicable diseases is increasing.^2^ These non-communicable diseases include diabetes,^3^ hypertension and cardiovascular diseases,^4^ chronic respiratory diseases,^5^ chronic renal diseases,^6^ mental and substance use disorders,^7^ and cancers.^8^

Although the transition of disease burden has predominantly included shifts from infectious diseases to non-communicable diseases globally, studies in South Africa and other regions across sub-Saharan Africa have reported a convergence of infectious diseases and non-communicable diseases.^9^, ^10^, ^11^, ^12^, ^13^, ^14^ This convergence could be linked to the ageing HIV-positive population in these regions, the associated increasing burden of non-communicable diseases among these individuals, and the hastening effect of HIV on non-communicable disease acquisition.^15^ Managing the convergence of diseases is of even greater concern since the beginning of the COVID-19 pandemic because poorly controlled multimorbidity has been associated with an increased risk of severe outcomes from COVID-19.^16^, ^17^ Moreover, an increase in ageing among people living with HIV as a result of the success of antiretroviral therapy has seen a subsequent increase in non-communicable diseases in this group, resulting in recognition of the need for integrated infectious disease programmes and non-communicable disease care and prevention programmes to avoid a loss of health gains made through antiretroviral therapy.^10^, ^14^, ^18^, ^19^ The UN Sustainable Development Goal number 3, which aims to ensure healthy lives and promote wellbeing for people at all ages, advocates for the integration of infectious disease and non-communicable disease prevention and treatment.^20^ However, the extent to which the health needs of individuals with multiple conditions overlap within individuals and communities, and thus the most efficient and effective approach of designing a health-systems response, is not well established.

**Research in context**

**Evidence before this study**

We searched PubMed from database inception to March 15, 2022, using the search terms “non-communicable diseases”, “met health needs”, “unmet health needs”, “prevalence”, “HIV”, “diabetes”, “hypertension”, “Africa”, “sub-Saharan Africa”, and “South Africa” for articles published in English. This search revealed that, in the global context, there is an increasing burden of non-communicable diseases and the burden of communicable diseases continues to be high in Africa relative to other regions. This convergence of infectious and non-communicable diseases in low-resource settings has led to multiple calls for the integration of health systems that are currently independent to address multimorbidity. Health-systems data and reports on global burden of diseases show the extent of the problem. However, patient-level data that define the detailed health needs of individuals for both communicable and non-communicable diseases are scarce. Furthermore, the varied nature of non-communicable diseases and the widely varying health needs of people with these conditions have resulted in multiple approaches to defining individual and community-level health needs. Thus, the complexity of multimorbidity is a barrier to the development of unified approaches to define gaps in the health system and the design of interventions to address these gaps.

**Added value of this study**

This analysis introduces a simple framework for the definition of health needs that is applicable to infectious and non-communicable diseases. Use of this framework to analyse data from a large, population-based, multimorbidity study provides a comprehensive understanding of the met and unmet health needs of individuals living with HIV, diabetes, or hypertension in an HIV hyperendemic setting in a largely rural region of South Africa from 2018 to 2020. This analytical approach allows for the consideration of these health states individually and in combination. Furthermore, this approach allows for analysis at both the individual level and the community level. Applied to one community in rural South Africa, it shows that the health needs of people with HIV are generally well met, whereas the health needs of people with non-communicable diseases are poorly met by existing health systems. The analysis also shows that, within the community, areas of disease-specific and disease-non-specific health needs can be identified.

**Implications of all the available evidence**

Taken together with global findings that show the increasing burden and health needs of people living with non-communicable diseases, the framework introduced by this analysis will help countries to assess their health programmes, identify priority areas for intervention, and consider integrated approaches to communicable and non-communicable disease management. Within South Africa, the findings of this analysis suggest that the need for improved diagnosis, care, and disease control for people with diabetes and hypertension can be addressed by adapting the health systems that successfully meet the health needs of people living with HIV. The increasing non-communicable disease burden in low-income and middle-income countries, alongside ongoing communicable disease epidemics, indicates the need for improved integrative health care and calls for creative and affordable approaches to disease diagnosis and management.

In this analysis, we used results from the Vukuzazi study, a multimorbidity survey conducted in rural South Africa^9^ to assess the health needs of individuals and communities in rural KwaZulu-Natal and describe a needs scale that assesses health needs for infectious diseases and non-communicable diseases.

## Methods

### Study design and participants

This analysis used data collected during the Vukuzazi study, a large, community-based, cross-sectional multimorbidity survey conducted in the uMkhanyakude district in rural KwaZulu-Natal, South Africa, that were collected between May 25, 2018, and March 13, 2020. The methods of this survey have been described previously^9^, ^21^ and are provided in appendix 2 (pp 8–11). Briefly, the study area covered a 482 km^2^ radius within the demographic and health surveillance area of the Africa Health Research Institute (AHRI) in KwaZulu-Natal. The area has high HIV prevalence and antiretroviral therapy has been available through public health clinics since 2004. All current 36 097 residents aged 15 years or older within the survey area were considered eligible for the survey and were invited to mobile health camps to complete a health survey, multimorbidity screening, and collection of samples for biobanking (appendix pp 10–11). All participants who provided written informed consent and were enrolled in the survey were included in this analysis (appendix p 12). Female sex, older age, being unemployed, having lower socioeconomic and educational status, and being a resident of a rural area were characteristics that were over-represented among participants compared with eligible non-participants (appendix p 13).^21^

All participants of Vukuzazi were also members of an ongoing demographic and health surveillance programme, which has been described elsewhere.^21^ Briefly, the programme has conducted annual household, demographic, and health surveys every year since 2017 and includes a clinic surveillance system (ClinicLink) that provides clinic attendance data for the 11 primary health facilities in the surveillance area.^22^ For this analysis, data from the Vukuzazi survey and the demographic and health surveillance surveys were linked with a unique identifier that was common to both studies.

The Vukuzazi study was approved by the University of KwaZulu-Natal Biomedical Research Ethics Committee and the institutional review board of Mass General Brigham (Boston, MA, USA). Written consent for all study procedures and linkage to health and demographic surveillance information was obtained from all participants at mobile health camps.

### Procedures

Questionnaires were used to assess the diagnosed health conditions and treatment history of an individual for each disease (ie, HIV, diabetes, and hypertension) at the mobile health camps (appendix pp 10–11, 17–28). Anthropometric measures and blood pressure were collected according to the WHO STEPwise approach to Surveillance (STEPS) protocol. Blood samples were collected for assessment of glycated haemoglobin (HbA_1c_) and HIV immunoassay testing. Positive HIV immunoassay tests were followed by a reflex HIV-1 RNA viral load assessment. Typical or expected results were reported by telephone call or text message, whereas participants with unexpected results received an at-home visit for further assessment, communication of results, and referral into the health system for care.^21^

Data from the most recent annual surveillance before Vukuzazi enrolment for each participant (ie, 2017–19 depending on date of enrolment) were used for this analysis. Self-reported data, including socioeconomic status, perceived overall health, residence status, and geolocation, are collected regularly as part of the general health and sociodemographic questionnaire (administered by the Population Intervention Programme). The number of clinic visits individuals made in the past 12 months before Vukuzazi enrolment was obtained through linkage with the ClinicLink system.^22^ Participants included in the study were geolocated to their homes with a geographic information system.^23^ The Vukuzazi study used self-report to collect sex data (options were male or female).

We incorporated concepts that had previously been used to define unmet health needs and the treatment care cascade^24^, ^25^ and defined five health states on the basis of parallel diagnostic criteria for each of the three chronic diseases included in this analysis. The five health states were free of the condition, diagnosed and optimally treated, diagnosed and suboptimally treated, diagnosed but not engaged in care, and undiagnosed but had a positive screening test in Vukuzazi (table 1).

**Table 1:** Health state definitions for HIV, diabetes, and hypertension

	**HIV**	**Diabetes**	**Hypertension**
Free of the condition	Immunoassay negative	No previous diagnosis of diabetes and HbA_1c_ ≤6·5%	No previous diagnosis of hypertension and blood pressure <140/<90 mm Hg
Diagnosed, engaged in care, and optimally treated	Known diagnosis of HIV, on treatment, and HIV viral load <40 copies per mL	Known diagnosis of diabetes, on treatment, and HbA_1c_ ≤6·5%	Known diagnosis of hypertension, on treatment, and blood pressure ≤140/≤90 mm Hg
Diagnosed, engaged in care, and suboptimally treated	Known diagnosis of HIV, on treatment, and HIV viral load >40 copies per mL	Known diagnosis of diabetes, on treatment, and HbA_1c_ >6·5%	Known diagnosis of hypertension, on treatment, and blood pressure ≥140/≥90 mm Hg
Diagnosed but not engaged in care	Known diagnosis of HIV, not on treatment, and HIV viral load >40 copies per mL	Known diagnosis of diabetes, not on treatment, and HbA_1c_>6·5%	Known diagnosis of hypertension, not on treatment, and blood pressure ≥140/≥90 mm Hg
Undiagnosed but had a positive screening test in the Vukuzazi study	No previous diagnosis of HIV, immunoassay positive, and HIV viral load >40 copies per mL	No previous diagnosis of diabetes and HbA_1c_ ≥6·5%	No previous diagnosis of hypertension and blood pressure ≥140/≥90 mm Hg

HBA_1c_=glycated haemoglobin.

**Figure 1:**
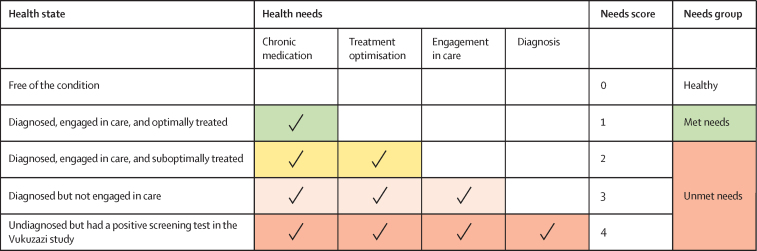
Framework for understanding the relationship between health states, health needs, needs scores, and needs groups

We developed a novel framework to relate these five health states to their respective health-system needs. The health-system needs of each health state were captured by a needs score in which the lowest score (0) represented an absence of disease and thus no immediate needs from the health system and the highest score (4) represented individuals who had the highest health needs and required diagnosis, engagement in care (defined as visiting a health-care facility for treatment of a disease), treatment optimisation (defined as receiving treatment that results in reaching optimal therapeutic targets for a disease), and provision of chronic medication (figure 1). Participants were assigned needs scores based on their health state and associated health needs. Needs scores for individuals with a disease were then separated into two needs groups: met health needs (needs score 1) and unmet health needs (needs score 2*–*4). Participants who were diagnosed, engaged in care, and optimally treated had an associated health need for chronic medication, were assigned a needs score of 1, and were included in the met needs group. Participants who were diagnosed, engaged in care, and suboptimally treated had an additional health need of treatment optimisation, were assigned a needs score of 2, and were included in the unmet health needs group. Participants who were diagnosed but not engaged in care had an additional health need of engagement in care, were assigned a needs score of 3, and were included in the unmet health needs group. Participants who were undiagnosed and had a positive screening test in the Vukuzazi study had all health needs, including the need for diagnosis, were assigned a needs score of 4, and were included in the unmet health needs group. Need scores were calculated for individual diseases and for all three diseases combined. In the combined analysis, individuals with more than one disease were assigned the needs score representing their highest need.

### Geospatial analysis

Having previously observed little data on the overlapping prevalence of HIV, diabetes, and hypertension within KwaZulu-Natal,^9^ we sought to assess the geospatial distribution of health needs for these three conditions in the demographic surveillance area. Data visualisation analysis of the distribution of health needs for each condition (ie, HIV, diabetes, and hypertension) and for all three diseases combined were generated with continuous surface maps of the prevalence distribution of each need. Spatial interpolations were generated with a standard Gaussian kernel interpolation method (with a search radius of 3 km), which has been used and validated in this population for mapping multiple HIV outcomes in the study area.^25^ Maps were created with ArcGIS Pro version 3.1.

### Statistical analysis

We calculated the proportion of participants with each need score by disease and for all diseases combined. We then compared the descriptive features of individuals within each combined need score using Pearson's χ^2^ test or the Kruskal-Wallis rank-sum test. Due to the descriptive nature of the research and the small proportion of missingness we used complete case analysis to describe the data. Statistical analyses were done in R version 4.2.1.

### Role of the funding source

The funders of the analysis had no role in study design, collection of data, data analysis, interpretation of data, or writing or editing of the manuscript.

## Results

**Figure 2:**
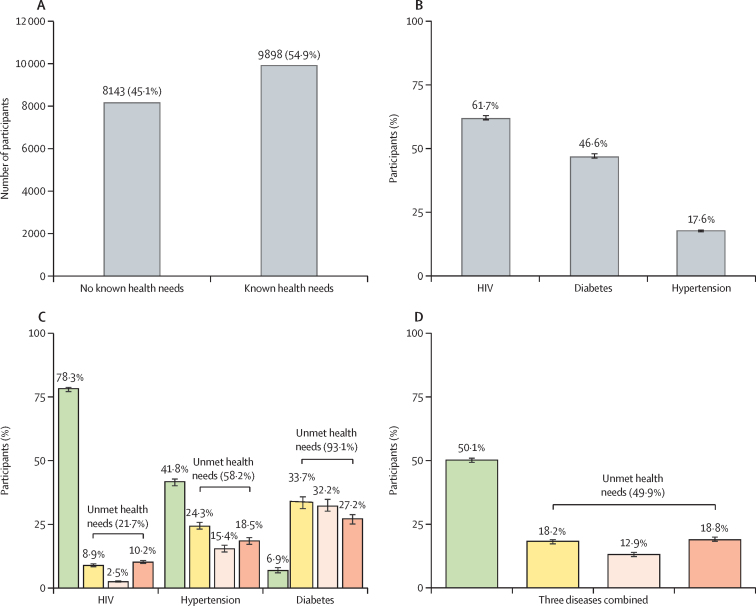
Distribution of health needs in the Vukuzazi cohort for participants with HIV, diabetes, or hypertension (A) Total number of participants with no health needs identified and with health needs identified. (B) Disease distribution among individuals with health needs identified. (C) Distribution of met and unmet health needs for individual chronic health states. (D) Distribution of met and unmet health needs for all three conditions combined (ie, HIV, diabetes, and hypertension). Green represents needs score 1 (ie, diagnosed with a well controlled condition), yellow represents needs score 2 (ie, diagnosed with a suboptimally controlled condition), light pink represents needs score 3 (ie, diagnosed but not engaged in care), and pink represents needs score 4 (ie, undiagnosed with an uncontrolled condition). Black error bars indicate 95% CIs.

Of the 18 041 individuals who enrolled in the Vukuzazi study, 9898 (54·9%) had at least one of the three health conditions measured (figure 2A). 12 229 (67·8%) of 18 041 participants were female and 5812 (32·2%) were male. Of the individuals with health conditions, 6096 (61·7%) had HIV, 4063 (46·6%) had hypertension, and 1737 (17·6%) had diabetes (figure 2B). The total number of participants with no health needs identified was 8143 (45·1%) of 18 041.

Distribution of sex, age, BMI, perceived general health state, number of clinic visits in the past year, distance to nearest clinic, residence location, socioeconomic status, drinking status, and household size differed between people with no health needs and people with different health needs scores (table 2). Health needs varied between age categories. For example, participants aged 25–44 years represented the largest proportion of people with well controlled chronic disease (needs score 1) and undiagnosed chronic disease (needs score 4), whereas participants aged 45–64 years represented the largest proportion of people with suboptimally controlled chronic disease (needs score 2) and chronic disease that was diagnosed but not treated (needs score 3).

**Table 2:** Demographic and socioeconomic data disaggregated by health needs

		**Overall**	**No health need (needs score 0)**	**Diagnosed with a well controlled condition (needs score 1)**	**Diagnosed with a suboptimally controlled condition (needs score 2)**	**Diagnosed but not engaged in care (needs score 3)**	**Undiagnosed with an uncontrolled condition (needs score 4)**	p value*
Sex		..	..	..	..	..	..	<0·0001
	Male	5812/18 041 (32·2%)	3507/8143 (43·1%)	973/4956 (19·6%)	391/1802 (21·7%)	345/1282 (26·9%)	596/1858 (32·1%)	..
	Female	12 229/18 041 (67·8%)	4636/8143 (56·9%)	3983/4956 (80·4%)	1411/1802 (78·3%)	937/1282 (73·1%)	1262/1858 (67·9%)	..
Age, years		..	..	..	..	..	..	<0·0001
	15–24	4962/18 041 (27·5%)	4152/8143 (51·0%)	375/4956 (7·6%)	82/1802 (4·6%)	59/1282 (4·6%)	294/1858 (15·8%)	..
	25–44	6008/18 041 (33·3%)	2336/8143 (28·7%)	2328/4956 (47·0%)	367/1802 (20·4%)	284/1282 (22·2%)	693/1858 (37·3%)	..
	45–64	4595/18 041 (25·5%)	1104/8143 (13·6%)	1626/4956 (32·8%)	751/1802 (41·7%)	550/1282 (42·9%)	564/1858 (30·4%)	..
	65 or older	2476/18 041 (13·7%)	551/8143 (6·8%)	627/4956 (12·7%)	602/1802 (33·4%)	389/1282 (30·3%)	307/1858 (16·5%)	..
BMI		..	..	..	..	..	..	<0·0001
	Typical (18·5–24 kg/m^2^)	7053/17 842 (39·5%)	4058/8081 (50·2%)	1726/4909 (35·2%)	428/1758 (24·3%)	265/1257 (21·1%)	576/1837 (31·4%)	..
	Underweight (<18·5 kg/m^2^)	857/17 842 (4·8%)	528/8081 (6·5%)	195/4909 (4·0%)	47/1758 (2·7%)	33/1257 (2·6%)	54/1837 (2·9%)	..
	Overweight (25–30 kg/m^2^)	4048/17 842 (22·7%)	1660/8081 (20·5%)	1236/4909 (25·2%)	448/1758 (25·5%)	297/1257 (23·6%)	407/1837 (22·2%)	..
	Obese (>30 kg/m^2^)	5884/17 842 (33·0%)	1835/8081 (22·7%)	1752/4909 (35·7%)	835/1758 (47·5%)	662/1257 (52·7%)	800/1837 (43·5%)	..
Perceived general health (assessed via the PIP survey)		..	..	..	..	..	..	<0·0001
	Poor to fair	2192/15 912 (13·8%)	494/6780 (7·3%)	712/4578 (15·6%)	478/1685 (28·4%)	293/1210 (24·2%)	215/1659 (13·0%)	..
	Good	8758/15 912 (55·0%)	3591/6780 (53·0%)	2694/4578 (58·8%)	908/1685 (53·9%)	657/1210 (54·3%)	908/1659 (54·7%)	..
	Very good	4962/15 912 (31·2%)	2695/6780 (39·7%)	1172/4578 (25·6%)	299/1685 (17·7%)	260/1210 (21·5%)	536/1659 (32·3%)	..
Any clinic visits in the past year		9561/18 041 (53·0%)	2925/8143 (35·9%)	3824/4956 (77·2%)	1302/1802 (72·3%)	725/1282 (56·6%)	785/1858 (42·2%)	<0·0001
Number of clinic visits in the past year		..	..	..	..	..	..	<0·0001
	1	2068/9561 (21·6%)	1242/2925 (42·5%)	301/3824 (7·9%)	151/1302 (11·6%)	152/725 (21·0%)	222/785 (28·3%)	..
	2–4	3084/9561 (32·3%)	1105/2925 (37·8%)	1153/3824 (30·2%)	324/1302 (24·9%)	230/725 (31·7%)	272/785 (34·6%)	..
	5 or more	4409/9561 (46·1%)	578/2925 (19·8%)	2370/3824 (62·0%)	827/1302 (63·5%)	343/725 (47·3%)	291/785 (37·1%)	..
Distance to nearest clinic, km		2·63 (1·52–4·07)	2·75 (1·62–4·22)	2·46 (1·47–3·85)	2·53 (1·42–4·01)	3·29 (2·08–4·45)	2·27 (1·34–3·61)	<0·0001
Smoking status		..	..	..	..	..	..	<0·0001
	Never	16 573/18 024 (91·7%)	7383/8126 (90·9%)	4622/4956 (93·3%)	1692/1802 (93·9%)	1168/1282 (91·1%)	1708/1858 (91·9%)	..
	Ex-smoker	150/18 024 (0·8%)	58/8126 (0·7%)	46/4956 (0·9%)	19/1802 (1·1%)	16/1282 (1·2%)	11/1858 (0·6%)	..
	Current smoker	1301/18 024 (7·2%)	685/8126 (8·4%)	288/4956 (5·8%)	91/1802 (5·0%)	98/1282 (7·6%)	139/1858 (7·5%)	..
Drinking status		..	..	..	..	..	..	<0·0001
	Never	15 752/18 024 (87·4%)	7009/8126 (86·3%)	4409/4956 (89·0%)	1627/1802 (90·3%)	1125/1282 (87·8%)	1582/1858 (85·1%)	..
	No drinking in the past 12 months	306/18 024 (1·7%)	154/8126 (1·9%)	73/4956 (1·5%)	28/1802 (1·6%)	23/1282 (1·8%)	28/1858 (1·5%)	..
	Drinking in the past 12 months	1966/18 024 (10·9%)	963/8126 (11·9%)	474/4956 (9·6%)	147/1802 (8·2%)	134/1282 (10·5%)	248/1858 (13·3%)	..
Household size		..	..	..	..	..	..	<0·0001
	Small to medium household (1–5 members)	12 662/18 041 (70·2%)	5355/8143 (65·8%)	3668/4956 (74·0%)	1360/1802 (75·5%)	920/1282 (71·8%)	1359/1858 (73·1%)	..
	Large household (>5 members)	5379/18 041 (29·8%)	2788/8143 (34·2%)	1288/4956 (26·0%)	442/1802 (24·5%)	362/1282 (28·2%)	499/1858 (26·9%)	..
Residence location		..	..	..	..	..	..	<0·0001
	Rural	11 436/17 985 (63·6%)	5430/8119 (66·9%)	2951/4940 (59·7%)	1049/1795 (58·4%)	1104/1280 (86·3%)	902/1851 (48·7%)	..
	Periurban	5599/17 985 (31·1%)	2342/8119 (28·8%)	1672/4940 (33·8%)	644/1795 (35·9%)	160/1280 (12·5%)	781/1851 (42·2%)	..
	Urban	950/17 985 (5·3%)	347/8119 (4·3%)	317/4940 (6·4%)	102/1795 (5·7%)	16/1280 (1·3%)	168/1851 (9·1%)	..
Socioeconomic status		..	..	..	..	..	..	<0·0001
	Low	6457/17 468 (37·0%)	2920/7909 (36·9%)	1868/4768 (39·2%)	626/1744 (35·9%)	465/1259 (36·9%)	578/1788 (32·3%)	..
	Middle	6043/17 468 (34·6%)	2762/7909 (34·9%)	1652/4768 (34·6%)	573/1744 (32·9%)	442/1259 (35·1%)	614/1788 (34·3%)	..
	High	4968/17 468 (28·4%)	2227/7909 (28·2%)	1248/4768 (26·2%)	545/1744 (31·3%)	352/1259 (28·0%)	596/1788 (33·3%)	..

Data are n/N (%) or median (IQR). PIP=Population Intervention Programme.

* All p values are Pearson's χ^2^, except Distance to nearest clinic, km, which is Kruskal-Wallis rank-sum test.

9932 (55·7%) of 17 842 participants were overweight or obese (ie, BMI >25 kg/m^2^; table 2). These individuals were under-represented among those without health needs (3495 [43·2%] of 8081 participants) and over-represented among those with health needs: 2988 (60·9%) of 4909 participants had needs score 1, 1283 (73·0%) of 1758 had needs score 2, 959 (76·3%) of 1257 had needs score 3, and 1207 (65·7%) of 1837 had needs score 4.

Despite having unmet health needs, the majority of participants with undiagnosed and uncontrolled diseases (needs score 4) perceived their health to be good or very good overall (table 2). Similarly, 1207 (71·6%) of 1685 participants who required optimisation of treatment (needs score 2) and 917 (75·8%) of 1210 participants who required engagement in care (needs score 3), and thus were collectively deemed to have a suboptimally controlled condition, reported their perceived health status as good or very good.

Many individuals with unmet health needs had visited a clinic in the year before engaging in the Vukuzazi study (table 2). Overall, 785 (42·2%) of 1858 participants who were undiagnosed with an uncontrolled condition (need score 4), 725 (56·6%) of 1282 participants who required engagement in care (need score 3), and 1302 (72·3%) of 1802 participants who required optimisation of treatment (need score 2) visited a clinic in the past year. 2287 (46·3%) of 4942 participants with unmet health needs had two visits or more in the previous year.

Individuals who lived in rural areas were over-represented among people who had diagnosed chronic disease but were not engaged in care (needs score 3). People with the furthest distance to the nearest clinic were similarly over-represented among this group.

Of the 9898 participants found to have a chronic health condition, the patterns of met and unmet health needs differed by individual disease. While 4775 (78·3%) of 6096 participants who were HIV-positive had their health needs met (ie, were diagnosed and on chronic medication for optimal disease control), only 120 (6·9%) of 1737 participants with diabetes and 1922 (41·8%) of 4603 participants with hypertension had their health needs fully met (figure 2C). Unmet health needs for individuals with HIV were predominantly driven by the need for treatment optimisation (need score 2; 543 [8.9%] of 6096) and diagnosis (need score 4; 624 [10·2%]), with only 154 (2·5%) of participants with HIV requiring engagement in care (need score 3).

By contrast, for hypertension and diabetes, all three unmet needs, including engagement in care, contributed substantially to the high levels of unmet health needs. 1617 (93·1%) of 1737 people who screened positive for diabetes, 2681 (58·2%) of 4603 people who screened positive for hypertension, and 1321 (21·7%) of 6096 people who screened positive for HIV had unmet health needs (figure 2C). The need for diagnosis (need score 4) was greater for individuals with diabetes (472 [27·2%] of 1737) and hypertension (852 [18·5%] of 4603) than HIV (624 [10·2%] of 6096; appendix pp 14–15). Although 1145 (65·9%) of 1737 participants with diabetes and 1829 (39·7%) of 4603 participants with hypertension were aware of their diagnosis, they either received suboptimal treatment (need score 2) or were not initially engaged in care (need score 3; figure 2C). 1145 (65·9%) of 1737 participants who knew they had diabetes and 1829 (39·7%) of 4603 participants who knew they had hypertension required either engagement in care (560 [32·2%] with diabetes and 710 [15·4%] with hypertension) or optimisation of treatment (585 [33·7%] with diabetes and 1119 [24·3%] with hypertension).

When we assessed the health needs of the population for all three disease conditions combined, we found that of the 9898 (54·9%) of 18 041 participants who had at least one of the three health conditions, 4956 (50·1%) had their health needs met and 4942 (49·9%) had at least one unmet health need (figure 2D). Among those 4942 participants with unmet health needs, 1802 (36·5%) were diagnosed and on treatment that required optimisation (need score 2), 1282 (25·9%) were diagnosed but not engaged in care (need score 3), and 1858 (37·6%) were undiagnosed and were therefore in need of further diagnostic testing, engagement in care, optimisation of treatment, provision of chronic medication, and routine monitoring (need score 4; figure 2D; appendix p 14).

Health needs also varied by age and multimorbidity. 541 (11·5%) of 4684 participants with HIV only had a need for diagnosis, compared with 303 (29·9%) of 1015 of participants with HIV and comorbid hypertension and 86 (48·0%) of 179 participants with HIV and comorbid diabetes (appendix p 16). Overall, 462 (33·1%) of 1397 participants with HIV and a comorbid non-communicable disease required a diagnosis. Younger participants had the greatest need for diagnosis; 34 (66·7%) of 51 participants aged 15–29 years with comorbid HIV and hypertension needed a diagnosis compared with 178 (41·1%) of 433 participants aged 30–49 years and 91 (17·1%) of 531 participants aged 50 years or older with the same combination of conditions.

**Figure 3:**
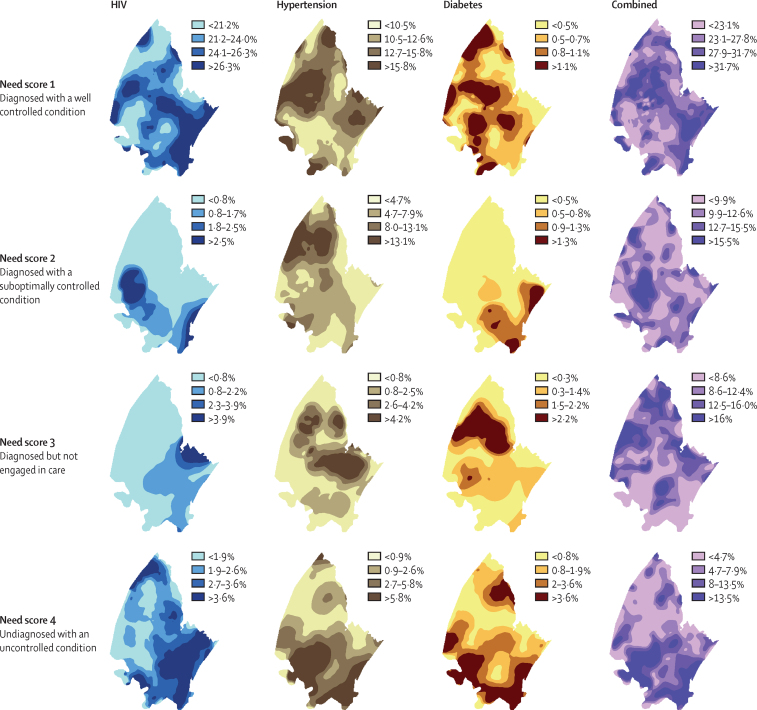
Geospatial distribution of health needs for HIV, hypertension, and diabetes individually and for all three chronic conditions combined

In our geospatial analysis, needs score 1 was widely distributed throughout the demographic surveillance area (figure 3), indicating that the need for chronic medication is present across the entire area for all three conditions. By contrast, needs scores 2 and 3 were specifically concentrated in more rural areas of the demographic surveillance area for all three conditions. Specifically, the need for optimisation of treatment for hypertension and the need for engagement in care for hypertension and diabetes were concentrated in the northern part of the surveillance area; the need for optimisation of treatment for diabetes was highest in the south-eastern part of the surveillance area. Needs scores 2 and 3 had low density in the southern-eastern part of the demographic surveillance area, the most densely populated region, whereas needs score 4 overlapped for all three conditions within this region, indicating a possible target area for diagnostic interventions (figure 3).

## Discussion

Using data from a large, community-based, cross-sectional multimorbidity survey in rural KwaZulu-Natal, South Africa, we assessed the complex health needs of individuals and communities and proposed and implemented a health-needs framework to conceptualise the met and unmet health needs of communities that are affected by the overlapping infectious disease and non-communicable disease epidemics in the country. The framework allows for establishment of similar health needs across chronic disease and promotes comparison between individuals with different health needs via sociodemographic and other health determinants. In our cohort in rural South Africa, we found that approximately half of people living with chronic disease in this community have unmet health needs. Use of this health-needs framework also allows for geographical visualisations that show colocalisation of individuals with undiagnosed infectious diseases and non-communicable diseases. Geospatial data visualisation by health needs also shows that analysing populations by their health needs provides useful disaggregation that is obscured when people with a specific condition are analysed in a group without regard to their other health needs. Our framework shows that analysing chronic disease separately and implementing public health approaches independently misses the opportunity for integration of communicable and non-communicable disease chronic care. Consideration should be given to health systems that are designed to address multiple health conditions and serve people with multiple chronic diseases.

More than half of the individuals who engaged in community-based health screening had at least one health need for the diagnosis or management of HIV, diabetes, or hypertension, but the met or unmet status of these needs differed between HIV and non-communicable diseases. 78·3% of participants with HIV, who were widely distributed throughout the geospatial area, had a well controlled condition and were on antiretroviral therapy. This finding shows the successful public health response to HIV in its ability to diagnose, optimally treat, and monitor people with a chronic infection across a large rural area. However, it also highlights the contrast between HIV and non-communicable disease responses; 93·1% of people who screened positive for diabetes and 58·2% of people who screened positive for hypertension have unmet health needs in this same community.

The lack of non-communicable disease control is similar to results reported in other studies in the region.^26^, ^27^, ^28^ For example, the South African National Health and Nutrition Examination Survey (SANHANES), which considered the prevalence of unmet health needs in South Africa, estimated that 91·5% of people with hypertension and 80·6% of people with diabetes had an unmet health need.^26^, ^27^ Although the SANHANES study showed that older individuals and those with obesity were more likely to have undiagnosed or poorly controlled diabetes, our analysis shows that younger participants (aged 15–29 years) were more likely to require diagnosis of comorbid HIV and hypertension (66·7%) than older participants (aged 30–49 years [41·1%] or aged >50 years [17·1%]; appendix p 16). With obesity representing an emerging problem across all age groups in South Africa and our analysis reporting associations between increased BMI and unmet health needs, the need for optimal diagnosis and treatment of non-communicable diseases in people who are overweight or obese is highlighted. Smaller studies in the province of Mpumalanga also reported high prevalence of uncontrolled hypertension (54·2–56·8%).^28^ These studies assessed the health needs of people with hypertension and diabetes, but our analysis has provided a framework for the assessment of these health needs simultaneously with HIV.

This analysis revealed a discrepancy between the ability of the South African health system to respond to the health needs of people with communicable diseases and the health needs of people with non-communicable diseases; 11·5% of participants with HIV only required a diagnosis whereas 33·1% of participants with HIV and a comorbid non-communicable disease required a diagnosis. Our results highlight the substantial need for improved non-communicable disease care in rural South Africa. With health systems currently reaching a wide target population for HIV care, creative adaptation of existing health programmes and frameworks could be successful in treating multiple chronic diseases concurrently.

Unmet health needs also varied by disease and geospatial location in the community. For HIV, most participants with unmet health needs required a diagnosis (10·2%) or optimisation of treatment (8·9%). Few participants required engagement in care, despite a known diagnosis. These data indicate that individuals who have been diagnosed with HIV have mostly been engaged in care and are receiving optimal antiretroviral therapy. Conversely, for non-communicable diseases, people who knew they had diabetes or hypertension required either engagement in care or optimisation of treatment. These differences could partly reflect difficulties in accessing care as individuals requiring engagement in care tended to live furthest from a clinic and were more likely to live in a rural setting compared with those with other need scores. By contrast, the need for treatment optimisation (need score 2) was higher in older people and people with higher BMI. Individuals with this health need were predominantly aged 45 years or older and were typically overweight or obese.

The association between increased BMI and suboptimal treatment of hypertension, diabetes, or other chronic diseases has been reported in other studies in which links between obesity, treatment-resistant hypertension, and altered pharmacological activity of drugs have been reported, with use of multiple agents suggested.^29^ Collectively, these data support the implementation of decentralised, patient-centred treatment programmes that consider patient variables such as barriers to health-care access, BMI, and age when providing treatment for non-communicable diseases.

The need for diagnosis (need score 4) was greater for individuals with diabetes and hypertension than HIV. The high prevalence of undiagnosed diabetes (45·4%) and hypertension (48·7%) in South Africa has also been reported in the SANHANES study.^26^, ^27^ When disaggregated by age, participants in each age group needed a diagnosis for HIV and comorbid diabetes or hypertension, indicating a universal need for integration of HIV and non-communicable disease care. Individuals with a need for diagnosis for all three conditions were concentrated in the southern part of the surveillance area, the most densely populated region in this analysis. Collectively, these data show a need to improve access to testing for non-communicable diseases. They also show an opportunity for targeted integrated interventions for non-communicable diseases and HIV in the demographic surveillance area, with more research required to establish whether these results are applicable to the country or region. Health-care facilities might have missed opportunities to address the health needs of people with a diagnosis requiring treatment optimisation (needs score 2) or engagement in care (need score 3), or even people who require a diagnosis (need score 4). The majority of these participants had visited a clinic in the area two or more times in the year before engaging in the Vukuzazi study, but still had unmet health needs at the time of the survey, which shows the need for improved, integrated primary health care.

Our analysis has several limitations. First, only three chronic disease conditions were considered. However, the proposed framework offers flexibility and can be extended to other conditions. Second, Vukuzazi only enrolled half of the eligible population, which might bias our description of health needs and their associations. The direction of bias is hard to anticipate based on the known demographic differences between the sampled and unsampled population because the health status of the unenrolled population is unknown.^9^ The Vukuzazi study enrolled more female participants than male participants and more older people than younger people, both of which could lead to overestimation of diabetes, hypertension, and their health needs. The under-representation of male participants highlights that they have fewer interactions with both community-based and routine health services, and that their health needs are poorly understood and require particular attention in the future. We acknowledge that people who screened positive for diabetes and hypertension required confirmatory testing before confirmation of diagnosis, and that this testing could rule out a disease requiring immediate treatment; thus, we might have overestimated the burden of undiagnosed disease.^30^ Finally, we acknowledge that ascribing the status of having no health needs to people who screen negative for disease is an oversimplification as it neglects the need for interventions targeting disease prevention, which might be crucial for optimal community health.

We have introduced a needs framework that allows for the analysis of health needs for multiple diseases concurrently despite their individualised prevention, treatment, and diagnostic parameters. This novel framework provides a way to conceptualise and measure individual and community health needs for people living in communities with high rates of infectious and non-communicable diseases. Applying this framework shows that approximately half of the people living with HIV, diabetes, or hypertension in a South African community have unmet health needs and that the unmet needs are particularly high in people living with non-communicable diseases. Furthermore, the granularity of this framework identifies unanticipated geospatial patterns of health-need distribution that could inform strategies for improving rural health, such as scheduled visits by mobile clinics for health checks, medication distribution, or chronic disease management. New approaches to addressing these unmet health needs are urgently required and we suggest that applying a health-needs framework could provide novel insights and guide the design of integrated, decentralised, and patient-centred programmes for the management of infectious diseases and non-communicable diseases.

The findings of this analysis suggest that in South Africa, health systems that have successfully met the needs of people living with HIV should be used to address the unmet needs of people living with hypertension and diabetes. With the burden of non-communicable diseases increasing globally, especially in low-income and middle-income countries where they occur alongside epidemics of communicable diseases, there is an urgent need for integrating primary health care and developing creative and affordable approaches to multidisease diagnosis and management.

## Data sharing

Data and related documents for the Vukuzazi study and for this analysis, including the study protocol, informed consent forms, de-identified participant data, and a data dictionary defining each field, can be accessed via the Africa Health Research Institute Data Repository (RDMServiceDesk@ahri.org) after publication upon approval of the proposed analyses by the Vukuzazi Scientific Steering Committee and completion of a data access agreement.

For **ArcGIS Pro** see http://www.esri.com

## Declaration of interests

We declare no competing interests.

## Supplementary Materials

isiZulu translation of the abstract
