# Supplementary material for: The met and unmet health needs for HIV, hypertension, and diabetes in rural KwaZulu-Natal, South Africa: analysis of a cross-sectional multimorbidity survey
Source: Lancet Glob Health. 2023 Aug 15;11(9):e1372–82. doi: 10.1016/S2214-109X(23)00239-5 (PMC10447220; doi:10.1016/S2214-109X(23)00239-5)
Supplement: isiZulu translation of the abstract [file mmc1.pdf]

THE LANCET
Global Health

Supplementary appendix 1

This translation in isiZulu was submitted by the authors and we reproduce it as supplied. It has not been peer reviewed. *The Lancet*'s editorial processes have only been applied to the original in English, which should serve as reference for this manuscript.

Lokhu kuhunyushwa ngolimi lwesiZulu kwethulwe ngababhali futhi siyikhiqiza kabusha njengoba kuhlinzekiwe. Akuzange kubuyekezwe ontanga. Izinqubo zokuhlela zeLancet zisetshenziswe kuphela koqobo lwesiNgisi, okufanele lusebenze njengesethenjwa salo mbhalo.

Supplement to: Singh U, Olivier S, Cuadros D, et al. The met and unmet health needs for HIV, hypertension, and diabetes in rural KwaZulu-Natal, South Africa: analysis of a cross-sectional multimorbidity survey. *Lancet Glob Health* 2023; **11:** e1372–82.

Isifingqo (Abstract)

Ingemuva (Background)

Ukuhlangana kwezifo ezithathelanayo nezingathathelwana (ama-NCD) eNingizimu Afrika kubangela inselele ezinhlelweni zezempilo. Lapha sisungula uhlaka lokulinganisa izidingo zezempilo ezihlangatsheziwe nezingafinyelelanga kubantu abaphila nezifo ezithathelanayo kanye nama-NCD.

Izindlela (Methods)

Sihlaziye imininingwane evela kubo bonke ababambiqhaza ocwaningweni lwezifo eziningi olusekelwe emphakathini oluhlinzeka ngokuhlolela i-HIV, umfutho wegazi ophakeme (HNTN) nesifo sikashukela (DM) kubo bonke abantu abasha kanye nabantu abadala abahlala endaweni egada abantu kanye nezempilo esifundeni saseMkhanyakude, KwaZulu-Natal, eNingizimu Afrika. Esimweni ngasinye, abantu babechazwa njengabangenazo izidingo zezempilo ezingahlangatshezwana nazo (ukungabikho kwesimo), bahlangabezana nesidingo sezempilo (isimo esilawulwa kahle), noma isidingo sezempilo esisodwa noma ngaphezulu esingahlangabeziwe (okuhlanganisa ukuxilonga, ukuzibandakanya ekunakekelweni, noma ukuthuthukiswa kokwelashwa). Sihlaziye izidingo zempilo ezihlangatsheziwe nezingafinyelelwanga zomuntu ngamunye nezihlanganisiwe, futhi saphenya ukusatshalaliswa kwazo kwe-geospatial.

Okutholakele (Findings)

Kubahlanganyeli abawu- 18,041, abawu-9,898 (55%) babenesimo okungenani esisodwa esingamahlalakhona. Abawu-4,942 (50%) balaba bantu babenesidingo okungenani esisodwa esingakafinyelelwa (amaphesenti angu-18 badinga ukuthuthukiswa kokwelashwa, u-13% badinga ukuzibandakanya ekunakekelweni, futhi u-19% badinga ukuxilongwa). Izidingo zezempilo ezingakafinyelelwa ziyahluka ngokwezifo: u-93% wabantu abane-DM, u-58% wabantu abane-HPTN kanye no-21% wabantu abane-HIV babe nezidingo zezempilo ezingakafinyelelwa. Ngokwe-geospatial, izidingo zezempilo ze-HIV ezihlangatsheziwe zisatshalaliswa kabanzi, izidingo zezempilo ezingahlangatshezwana nazo zinezindawo ezithile zokuqoqana kuyilapho isidingo sokuxilonga kuzo zontathu izimo sasibekwe ndawonye.

Ukuhunyushwa (Interpretation)

Nakuba abantu abaphila ne-HIV belawulwa kahle kakhulu, kunomthwalo omkhulu wezidingo zezempilo ezingahlangatshezwa kubantu abaphila ne-HPTN no-DM. Ukujwayela izibonelo zokunakekelwa kwe-HIV ukuze kuhlanganiswe izinsiza ze-HIV kanye ne-NCD kuseqhulwini.
