# Supplementary material for: The met and unmet health needs for HIV, hypertension, and diabetes in rural KwaZulu-Natal, South Africa: analysis of a cross-sectional multimorbidity survey
Source: Lancet Glob Health. 2023 Aug 15;11(9):e1372–82. doi: 10.1016/S2214-109X(23)00239-5 (PMC10447220; doi:10.1016/S2214-109X(23)00239-5)
Supplement: Supplementary appendix 2 [file mmc2.pdf]

THE LANCET
Global Health

Supplementary appendix 2

This appendix formed part of the original submission and has been peer reviewed. We post it as supplied by the authors.

Supplement to: Singh U, Olivier S, Cuadros D, et al. The met and unmet health needs for HIV, hypertension, and diabetes in rural KwaZulu-Natal, South Africa: analysis of a cross-sectional multimorbidity survey. *Lancet Glob Health* 2023; **11:** e1372–82.

Appendix

Contents

Strobe checklist 2
Vukuzazi study team 5
Supplemental methods 8
Figure S1. Cohort design, data collection and mobile health camp structure 10
Figure S2. Overview of recruitment of participants in the Vukuzazi study 12
Table S1. Differences between enrolled and unenrolled participants 13
Supplemental figures and tables 14
Table S2. Confidence interval data for figure 2 14
Table S3. Needs scores broken down by disease combinations. 16
Vukuzazi questionnaires 17
References 29

Strobe checklist

STROBE Statement—Checklist of items that should be included in reports of ***cross-sectional studies***

	Item No	Recommendation	
**Title and abstract**	1	(*a*) Indicate the study's design with a commonly used term in the title or the abstract	✓
		(*b*) Provide in the abstract an informative and balanced summary of what was done and what was found	✓
**Introduction**			
Background/rationale	2	Explain the scientific background and rationale for the investigation being reported	✓
Objectives	3	State specific objectives, including any prespecified hypotheses	✓
**Methods**			
Study design	4	Present key elements of study design early in the paper	✓
Setting	5	Describe the setting, locations, and relevant dates, including periods of recruitment, exposure, follow-up, and data collection	✓
Participants	6	(*a*) Give the eligibility criteria, and the sources and methods of selection of participants	✓
Variables	7	Clearly define all outcomes, exposures, predictors, potential confounders, and effect modifiers. Give diagnostic criteria, if applicable	✓
Data sources/ measurement	8*	For each variable of interest, give sources of data and details of methods of assessment (measurement). Describe comparability of assessment methods if there is more than one group	✓
Bias	9	Describe any efforts to address potential sources of bias	✓
Study size	10	Explain how the study size was arrived at	✓
Quantitative variables	11	Explain how quantitative variables were handled in the analyses. If applicable, describe which groupings were chosen and why	✓
Statistical methods	12	(*a*) Describe all statistical methods, including those used to control for confounding	✓
		(*b*) Describe any methods used to examine subgroups and interactions	✓
		(*c*) Explain how missing data were addressed	✓
		(*d*) If applicable, describe analytical methods taking account of sampling strategy	✓
		(*e*) Describe any sensitivity analyses	✓

**Results**			
Participants	13*	(a) Report numbers of individuals at each stage of study—eg numbers potentially eligible, examined for eligibility, confirmed eligible, included in the study, completing follow-up, and analysed	✓
		(b) Give reasons for non-participation at each stage	✓
		(c) Consider use of a flow diagram	✓
Descriptive data	14*	(a) Give characteristics of study participants (eg demographic, clinical, social) and information on exposures and potential confounders	✓
		(b) Indicate number of participants with missing data for each variable of interest	✓
Outcome data	15*	Report numbers of outcome events or summary measures	✓
Main results	16	(*a*) Give unadjusted estimates and, if applicable, confounder-adjusted estimates and their precision (eg, 95% confidence interval). Make clear which confounders were adjusted for and why they were included	✓
		(*b*) Report category boundaries when continuous variables were categorized	
		(*c*) If relevant, consider translating estimates of relative risk into absolute risk for a meaningful time period	✓
Other analyses	17	Report other analyses done—eg analyses of subgroups and interactions, and sensitivity analyses	✓
**Discussion**			
Key results	18	Summarise key results with reference to study objectives	✓
Limitations	19	Discuss limitations of the study, taking into account sources of potential bias or imprecision. Discuss both direction and magnitude of any potential bias	✓
Interpretation	20	Give a cautious overall interpretation of results considering objectives, limitations, multiplicity of analyses, results from similar studies, and other relevant evidence	✓
Generalisability	21	Discuss the generalisability (external validity) of the study results	✓
**Other information**			
Funding	22	Give the source of funding and the role of the funders for the present study and, if applicable, for the original study on which the present article is based	✓

*Give information separately for exposed and unexposed groups.

Note: An Explanation and Elaboration article discusses each checklist item and gives methodological background and published examples of transparent reporting. The STROBE checklist is best used in conjunction with this article (freely available on the Web sites of PLoS Medicine at http://www.plosmedicine.org/, Annals of Internal Medicine at http://www.annals.org/, and Epidemiology at http://www.epidem.com/). Information on the STROBE Initiative is available at www.strobe-statement.org.

Vukuzazi study team

Vukuzazi Team: Staff who significantly contributed to the implementation and conduct of Vukuzazi.

***** Denotes team members who were closely involved with the design, implementation and oversight of Vukuzazi.

**Name**	**Role**
*Deenan Pillay	Principal Investigator (2017-2019)
*Willem Hanekom	Principal Investigator (2019-present)
*Emily Wong	Co-Principal Investigator
*Mark Siedner	Co-Principal Investigator
*Olivier Koole	Co-Principal Investigator (2017-2019)
*Thumbi Ndung'u	Co-investigator
*Thandeka Khoza	Co-investigator (2019-present)
*Kobus Herbst	Co-investigator
*Kathy Baisley	Co-investigator
*Janet Seeley	Co-investigator
*Alison Grant	Co-investigator
*Resign Gunda	Programme Manager
*Ashmika Surujdeen	Study Coordinator
*Theresa Smit	Head: Diagnostic Research
*Dickman Gareta	Head: Research Data Management
*Day Munatsi	Head: Research Data Systems
*Ngcebo Mhlongo	Study Physician
*Sanah Bucibo	Lead Nurse
*Tshwaraganang Modise	Research Data Manager
*Stephen Olivier	Statistician
*Gregory Ording-Jespersen	Laboratory Data Supervisor
*Innocentia Mpofana	Diagnostic Laboratory Manager
*Jaco Dreyer	Senior Research Data Manager
*Siyabonga Nxumalo	Research Data Manager
*Khadija Khan	Biorepository Manager
*Zizile Sikhosana	Somkhele Laboratory Supervisor
*Sashen Moodley	Microbiology Laboratory Supervisor
*Hollis Shen	Head: Exploratory Research Division
Kennedy Nyamande	Pulmonology Consultant
Mosa Suleman	Pulmonology Consultant
Jaikrishna Kalideen	Radiologist
Ramesh Jackpersad	Radiologist
Kgaugelo Moropane	Radiographer
Boitsholo Mfolo	Radiographer
Khabonina Malomane	Radiographer
Hlolisile Khumalo	Nursing Manager
Nompilo Buthelezi	Training Coordinator
Nozipho Mbonambi	Professional Nurse
Hloniphile Ngubane	Professional Nurse
Thokozani Simelane	Professional Nurse
Khanyisani Buthelezi	Professional Nurse
Sphiwe Ntuli	Professional Nurse
Nombuyiselo Zondi	Professional Nurse

Siboniso Nene	Professional Nurse
Bongumenzi Ndlovu	Enrolled Nurse
Talente Ntimbane	Enrolled Nurse
Mbali Mbuyisa	Enrolled Nurse
Xolani Mkhize	Enrolled Nurse
Melusi Sibiya	Enrolled Nurse
Ntombiyenkosi Ntombela	Enrolled Nurse
Mandisi Dlamini	Enrolled Nurse
Hlobisile Chonco	Enrolled Nurse
Hlengiwe Dlamini	Enrolled Nurse
Doctar Mlambo	Enrolled Nurse
Nonhlanhla Mzimela	Enrolled Nurse
Zinhle Buthelezi	Enrolled Nurse
Zinhle Mthembu	Enrolled Nurse
Thokozani Bhengu	Enrolled Nurse
Sandile Mthembu	Enrolled Nurse
Phumelele Mthethwa	Enrolled Nurse
Zamashandu Mbatha	Enrolled Nurse
Welcome Petros Mthembu	Enrolled Nurse
Anele Mkhwanazi	Clinical Research Assistant Supervisor
Mandlakayise Zikhali	Clinical Research Assistant Supervisor
Phakamani Mkhwanazi	Clinical Research Assistant
Ntombiyenhlanhla Mkhwanazi	Clinical Research Assistant
Rose Myeni	Clinical Research Assistant
Fezeka Mfeka	Clinical Research Assistant
Hlobisile Gumede	Clinical Research Assistant
Nonceba Mfeka	Clinical Research Assistant
Ayanda Zungu	Clinical Research Assistant
Hlobisile Gumede	Clinical Research Assistant
Nonhlanhla Mfekayi	Clinical Research Assistant
Smangaliso Zulu	Clinical Research Assistant
Mzamo Buthelezi	Clinical Research Assistant
Senzeni Mkhwanazi	Clinical Research Assistant
Mlungisi Dube	Clinical Research Assistant
Philippa Mathews	Clinical Governance
Siphephelo Dlamini	AHRI Nursing Manager
Hosea Kambonde	IT Systems Developer
Lindani Mthembu	Information Technology Assistant
Seneme Mchunu	Information Technology Assistant
Sibahle Gumbi	Research Admin Assistant
Tumi Madolo	Research Data Manager
Thengokwakhe Nkosi	Driver
Sibusiso Mkhwanazi	Driver
Sibusiso Nsibande	Driver
Mpumelelo Steto	Driver
Sibusiso Mhlongo	Driver
Velile Vellem	Driver
Pfarelo Tshivase	Driver
Jabu Kwinda	Driver
Bongani Magwaza	General Worker

Siyabonga Nsibande	General Worker
Skhumbuzo Mthombeni	General Worker
Sphiwe Clement Mthembu	General Worker
Antony Rapulana	Laboratory Technologist
Jade Cousins	Laboratory Technologist
Thabile Zondi	Laboratory Technologist
Nagavelli Padayachi	Laboratory Technologist
Freddy Mabetlela	Laboratory Technologist
Simphiwe Ntshangase	Laboratory Technician/LIMS Administrator
Nomfundo Luthuli	Laboratory Technician
Sithembile Ngcobo	Laboratory Technologist
Kayleen Brien	Laboratory Technologist
Sizwe Ndlela	Laboratory Technician
Nomfundo Ngema	Laboratory Technician
Nokukhanya Ntshakala	Laboratory Technician
Anupa Singh	Laboratory Technician
Rochelle Singh	Laboratory Technician
Logan Pillay	Laboratory Technician
Kandaseelan Chetty	Laboratory Technician
Ashentha Govender	Laboratory Technician
Pamela Ramkalawon	Laboratory Research Technician
Nondumiso Mabaso	Laboratory Intern
Kimeshree Perumal	Laboratory Intern
Senamile Makhari	Biorepository Laboratory Technician
Nondumiso Khuluse	Biorepository Laboratory Technician
Nondumiso Zitha	Biorepository Research Assistant
Hlengiwe Khathi	Biorepository Research Assistant
Mbuti Mofokeng	Clinical Specimen Driver/Laboratory Assistant
Nomathamsanqa Majozi	Public Engagement
Nceba Gqaleni	Public Engagement
Hannah Keal	Communications
Phumla Ngcobo	Communications
Costa Criticos	Operational Oversight
Raynold Zondo	Operational Oversight
Dilip Kalyan	Operational Oversight
Clive Mavimbela	Operational Oversight
Anand Ramnanan	Procurement
Sashin Harilall	Grants Office

Supplemental methods

Additional methodological details

Study area

The current manuscript reports the results of an analysis of data from the Vukuzazi study, which was a cross-sectional multimorbidity survey, the methodology of which has been published in detail in Gunda et al (1) and is explained in further detail below. The Vukuzazi study was conducted in the uMkhanyakude district in rural KwaZulu-Natal between May 2018 and March 2020. The study area was based in the southern portion of the Africa health research institute demographic surveillance area and covered a 482km^2 radius. The majority of residents in the area represented rural dwellers.

Participant eligibility and recruitment:

Eligible participants were defined as all individuals ≥15 years of age residing in the southern portion of the Africa Health Research Institute demographic surveillance area. To be considered a resident, individuals needed to have slept at their residence for most nights during the preceding four months of the study. Demographic surveillance data was used to establish a list of eligible participants. Fieldworkers then visited households of eligible participants and provided written and verbal explanations on the Vukuzazi study and mobile health camps. Eligible participants were given a registered invitation to mobile health camps, either personally or via a proxy if they were not present at the time of the visit. Individuals with registered invitations could access mobile health camps on presentation of the invitation (Figure S1a).

Mobile health camps

Mobile health camps were established within a 2km radius of homesteads. A total of 78 mobile health camps were held. On arrival at health camps, eligible participants first provided informed consent. enrolment in the study required that individuals were firstly eligible and secondly they provided written informed consent at the mobile health camps.

Once enrolled, participants completed a detailed health questionnaire and progressed to provide anthropometric and health measurements. This was followed by collection of blood samples, rectal swabs and sputum specimens. Lastly participants completed chest X-rays and then exited the mobile health camp (Figure S1b). Participants with normal test results were contacted and informed via sms or telephonically. Participants with abnormal results were invited for re-evaluation and were given their result at additional mobile clinics. Participants with persistently abnormal results following re-evaluation were linked to care (Figure S1a and Figure S1B).

Informed consent:

On arrival at mobile health camps, potential participants were provided with further explanations on the study and requirements from participants. Assessment of individuals capacity to provide consent was also conducted. All participants provided written informed consent for disease testing, collection, storage and future use of specimens collected and stored in the Africa health research institute biorepository. Participants also provided

permission to be contacted for follow-up studies and with results of tests. Participants <18 years of age required assent from a parent or guardian prior to enrolment in the study.

Health questionnaire:

Detailed health questionnaires were administered by staff nurses at the mobile health camps. Questionnaires explored personal history of HIV, tuberculosis, diabetes mellitus and hypertension (detailed questionnaire is included at the end of this document).

Figure S1. Cohort design, data collection and mobile health camp structure

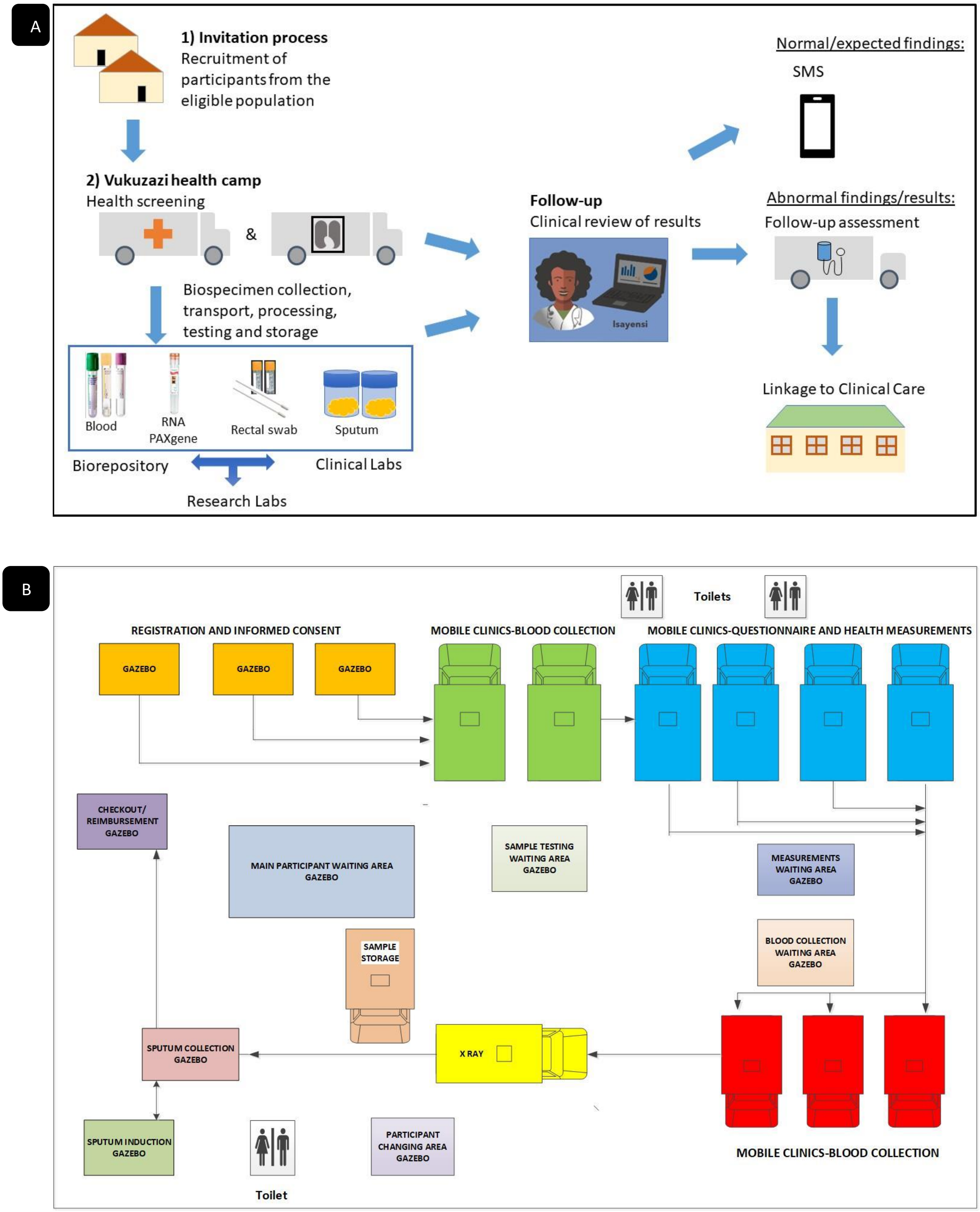

Figure S1. *Overview of components of the Vukuzazi program (a) and the design/layout of mobile health camps (b), taken from Gunda et al., 2022 (figure 2 and 3 respectively) (1). (a) Eligible participants (i.e., all community members ≥ 15 years of age living within the 482km^2 demographic surveillance area in the uMkhanyakude district were invited to attend mobile health camps. Invitations were delivered to households. Participants then visited mobile health camps, which were located within a*

2km radius of homesteads. A total of 78 mobile health camps were held. At mobile health camps participants provided informed consent, completed health related questionnaires, anthropometric measurements and provided samples (blood, rectal swabs and sputum) which were sent to clinical laboratories and/or stored in a biorepository for further assessment. Participants with normal or expected results received phone calls to communicate results. Whilst, participants with abnormal results were given follow-up assessments. If results were persistently abnormal after a second visit participants were linked to clinical care at their nearest health facility. (b) as part of mobile health camps, participants first provided informed consent at a gazebo, thereafter they progressed to the mobile clinics for blood collection followed by health questionnaires and anthropometric measurements. Following this, all eligible participants received chest x-rays and had their sputum collected prior to exiting the camp. *These images are re-published from Gunda et al., 2022 (1), which was published by Oxford University Press on behalf of the International Epidemiological Association as an Open Access article distributed under the terms of the Creative Commons Attribution License (https://creativecommons.org/licenses/by/4.0/), which permits unrestricted reuse, distribution, and reproduction in any medium, provided the original work is properly cited.*

Figure S2. Overview of recruitment of participants in the Vukuzazi study

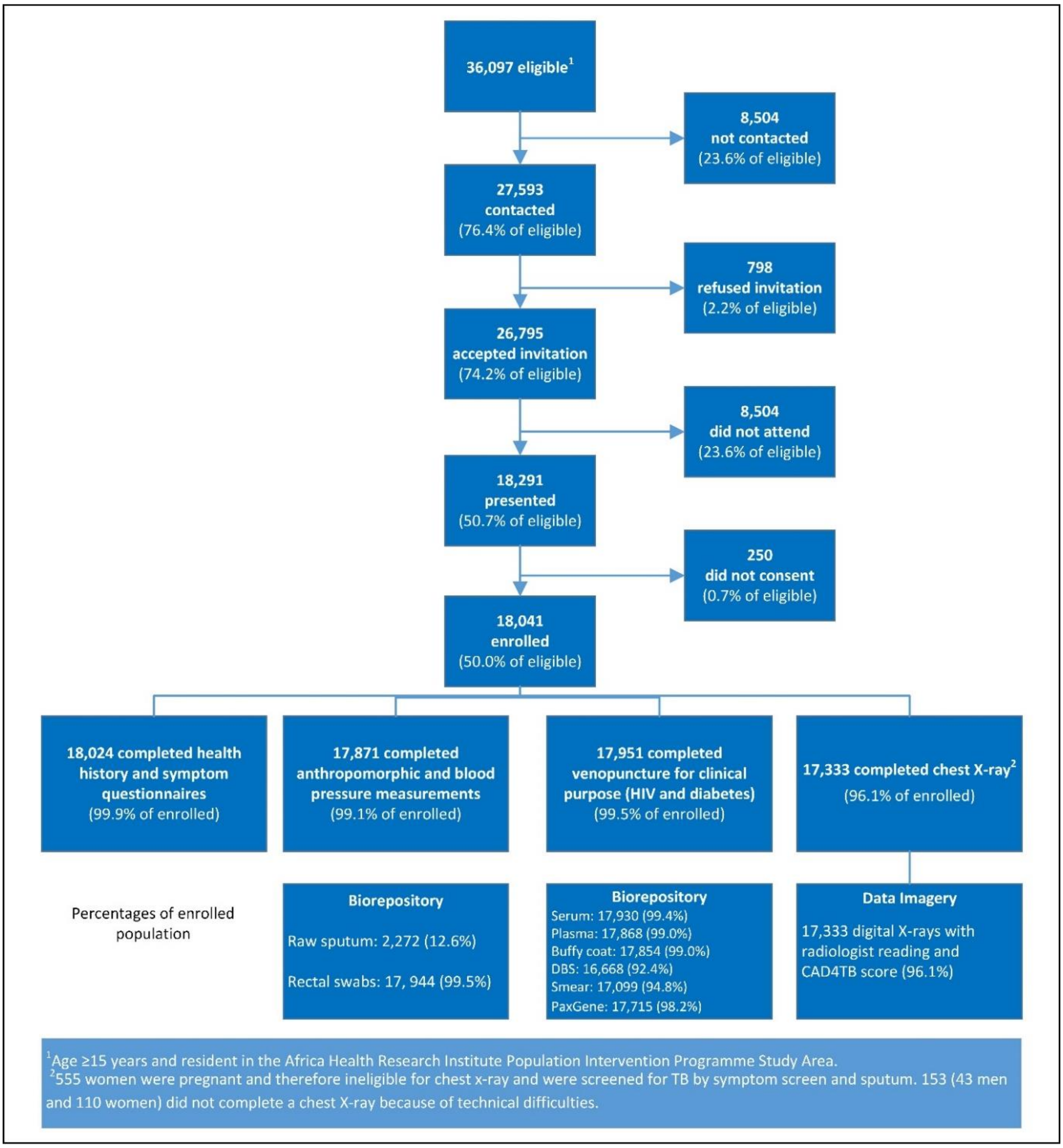

Figure S2 *Participant overview in the Vukuzazi study taken from Gunda et al., 2022 (1). Of the 36097 eligible participants at the time of the study, a total of 18041 were successfully enrolled in the study. The majority of participants completed all bio-sample, chest Xray and anthropometric assessments. Missing data did exist amongst participants.* *This image is re-published from Gunda et al., 2022 (1), which was published by Oxford University Press on behalf of the International Epidemiological Association as an Open Access article distributed under the terms of the Creative Commons Attribution License (https://creativecommons.org/licenses/by/4.0/), which permits unrestricted reuse, distribution, and reproduction in any medium, provided the original work is properly cited.*

Table S1. Differences between enrolled and unenrolled participants

**Characteristic**	**Vukuzazi participants N = 18,041[1]**	**Eligible population N = 36,097[1]**	**p-value[2]**
**Age group**			<0.001
15-24	4,977 (28%)	11,049 (31%)	
25-34	3,371 (19%)	7,854 (22%)	
35-44	2,634 (15%)	5,753 (16%)	
45-54	2,240 (12%)	4,192 (12%)	
55-64	2,353 (13%)	3,673 (10%)	
65+	2,466 (14%)	3,576 (10%)	
**Sex**			<0.001
Male	5,811 (32%)	14,965 (41%)	
Female	12,230 (68%)	21,132 (59%)	
**Education**			<0.001
None	4,532 (25%)	8,387 (24%)	
Less than secondary	6,737 (38%)	11,910 (33%)	
Secondary and above	6,666 (37%)	15,385 (43%)	
**Marital status**			<0.001
Single	3,757 (24%)	7,772 (26%)	
Married/Living as married	9,647 (62%)	19,408 (64%)	
Separated/divorced/widowed	2,155 (14%)	3,197 (11%)	
**Employment status**			<0.001
Unemployed	3,887 (57%)	7,307 (45%)	
Employed	2,979 (43%)	8,900 (55%)	
**Residence location**			<0.001
Urban	950 (5%)	2,849 (8%)	
Peri-Urban	5,599 (31%)	11,813 (33%)	
Rural	11,436 (64%)	21,299 (59%)	

Table S1 *Differences between the enrolled and unenrolled populations. This table is re-published from Gunda et al., 2022 (1), which was published by Oxford University Press on behalf of the International Epidemiological Association as an Open Access article distributed under the terms of the Creative Commons Attribution License (https://creativecommons.org/licenses/by/4.0/), which permits unrestricted reuse, distribution, and reproduction in any medium, provided the original work is properly cited.*

Supplemental figures and tables

Table S2. Confidence interval data for figure 2

Figure 2B: Disease distribution

Characteristic	N	N = 9,898[1]	95% CI[2]
HIVDiag	9,878	61.7	61% , 63%
HTNDiag	9,883	46.6	46% , 48%
DMDiag	9,879	17.6	17% , 18%

[1]%

[2]CI = Confidence Interval

Figure 2C-D: Needs scores for diseases

Characteristic	N	N = 9,898[1]	95% CI[2]
Max_detail	9,898		
Diagnosed and well controlled		50.1%	49% , 51%
Diagnosed suboptimally controlled		18.2%	17% , 19%
Diagnosed and not engaged		13.0%	12% , 14%
Undiagnosed and uncontrolled		18.8%	18% , 20%
HIV_detail	6,096		
Diagnosed and well controlled		78.3%	77% , 79%
Diagnosed suboptimally controlled		8.9%	8.2% , 9.7%
Diagnosed and not engaged		2.5%	2.2% , 3.0%
Undiagnosed and uncontrolled		10.2%	9.5% , 11%
HTN_detail	4,603		
Diagnosed and well controlled		41.8%	40% , 43%
Diagnosed suboptimally controlled		24.3%	23% , 26%
Diagnosed and not engaged		15.4%	14% , 17%
Undiagnosed and uncontrolled		18.5%	17% , 20%
DM_detail	1,737		
Diagnosed and well controlled		6.9%	5.8% , 8.2%
Diagnosed suboptimally controlled		33.7%	31% , 36%

Characteristic	N	N = 9,898[1]	95% CI[2]
Diagnosed and not engaged		32.2%	30% , 35%
Undiagnosed and uncontrolled		27.2%	25% , 29%

[1]%

[2]CI = Confidence Interval

Figure S3 Confidence interval data for figure 2.

Table S3. Needs scores broken down by disease combinations.

Table S3a: Overall need scores for individual and combinations of diseases.

HIVDiag	HTNDiag	DMDiag	Total	Need_1	Need_2	Need_3	Need_4
0	0	1	416	17 (4.1%)	79 (19%)	194 (46.6%)	126 (30.3%)
0	1	0	2428	905 (37.3%)	545 (22.4%)	464 (19.1%)	514 (21.2%)
0	1	1	936	48 (5.1%)	407 (43.5%)	268 (28.6%)	213 (22.8%)
1	0	0	4684	3566 (76.1%)	463 (9.9%)	114 (2.4%)	541 (11.5%)
1	0	1	179	9 (5%)	25 (14%)	59 (33%)	86 (48%)
1	1	0	1015	380 (37.4%)	205 (20.2%)	127 (12.5%)	303 (29.9%)
1	1	1	203	9 (4.4%)	71 (35%)	50 (24.6%)	73 (36%)

Table S3b: Need scores for individual and combinations of diseases amongst participants 15-29 years of age.

HIVDiag	HTNDiag	DMDiag	Total	Need_1	Need_2	Need_3	Need_4
0	0	1	52	5 (9.6%)	5 (9.6%)	31 (59.6%)	11 (21.2%)
0	1	0	202	17 (8.4%)	5 (2.5%)	45 (22.3%)	135 (66.8%)
0	1	1	3	0 (0%)	0 (0%)	2 (66.7%)	1 (33.3%)
1	0	0	1264	798 (63.1%)	148 (11.7%)	26 (2.1%)	292 (23.1%)
1	0	1	11	0 (0%)	0 (0%)	5 (45.5%)	6 (54.5%)
1	1	0	51	7 (13.7%)	4 (7.8%)	6 (11.8%)	34 (66.7%)

Table S3c: Need scores for individual and combinations of diseases amongst participants 30-49 years of age.

HIVDiag	HTNDiag	DMDiag	Total	Need_1	Need_2	Need_3	Need_4
0	0	1	85	1 (1.2%)	13 (15.3%)	35 (41.2%)	36 (42.4%)
0	1	0	327	80 (24.5%)	39 (11.9%)	84 (25.7%)	124 (37.9%)
0	1	1	55	4 (7.3%)	20 (36.4%)	18 (32.7%)	13 (23.6%)
1	0	0	2678	2111 (78.8%)	267 (10%)	84 (3.1%)	216 (8.1%)
1	0	1	85	2 (2.4%)	11 (12.9%)	23 (27.1%)	49 (57.6%)
1	1	0	433	126 (29.1%)	60 (13.9%)	69 (15.9%)	178 (41.1%)
1	1	1	46	1 (2.2%)	13 (28.3%)	14 (30.4%)	18 (39.1%)

Table S3d: Need scores for individual and combinations of diseases amongst participants ≥50 years of age.

HIVDiag	HTNDiag	DMDiag	Total	Need_1	Need_2	Need_3	Need_4
0	0	1	279	11 (3.9%)	61 (21.9%)	128 (45.9%)	79 (28.3%)
0	1	0	1899	808 (42.5%)	501 (26.4%)	335 (17.6%)	255 (13.4%)
0	1	1	878	44 (5%)	387 (44.1%)	248 (28.2%)	199 (22.7%)
1	0	0	742	657 (88.5%)	48 (6.5%)	4 (0.5%)	33 (4.4%)
1	0	1	83	7 (8.4%)	14 (16.9%)	31 (37.3%)	31 (37.3%)
1	1	0	531	247 (46.5%)	141 (26.6%)	52 (9.8%)	91 (17.1%)
1	1	1	157	8 (5.1%)	58 (36.9%)	36 (22.9%)	55 (35%)

Vukuzazi questionnaires

1) <u>History of NCDs</u>

History of Raised Blood Pressure		
**Question**	**Response**	**Code**
Have you ever had your blood pressure measured by a doctor or other health worker?	Yes 1<br>No 2 *If No, go to H4*	H1
Have you ever been told by a doctor or other health worker that you have raised blood pressure or hypertension?	Yes 1<br>No 2 *If No, go to H4*	H2a
Have you been told in the past 12 months?	Yes 1<br>No 2	H2b
In the past 6 months, have you seen a doctor or other health care worker about your high blood pressure?	Yes 1<br>No 2	H2c
In the past two weeks, have you taken any drugs (medication) for raised blood pressure prescribed by a doctor or other health worker?	Yes 1<br>No 2	H3
Have you ever seen a traditional healer for raised blood pressure or hypertension?	Yes 1<br>No 2 *If No, go to H6*	H4
Are you currently taking any herbal or traditional remedy for your raised blood pressure?	Yes 1<br>No 2	H5

History of Diabetes		
Have you ever had your blood sugar measured by a doctor or other health worker?	Yes 1<br>No 2 *If No, go to H10*	H6
Have you ever been told by a doctor or other health worker that you have raised blood sugar or diabetes?	Yes 1<br>No 2 *If No, go to H12*	H7a
Have you been told in the past 12 months?	Yes 1<br>No 2	H7b

In the past 6 months, have you seen a doctor or other health care worker about your raised blood sugar or diabetes?	Yes 1<br>No 2	H7c
In the past two weeks, have you taken any drugs (medication) for diabetes prescribed by a doctor or other health worker?	Yes 1<br>No 2	H8
Are you currently taking insulin for diabetes prescribed by a doctor or other health worker?	Yes 1<br>No 2	H9

Have you ever seen a traditional healer for diabetes or raised blood sugar?	Yes 1<br>No 2 *If No, go to H12*	H10
Are you currently taking any herbal or traditional remedy for your diabetes?	Yes 1<br>No 2	H11

**History of Raised Total Cholesterol**		
**Question**	**Response**	**Code**
Have you ever had your cholesterol (fat levels in your blood) measured by a doctor or other health worker?	Yes 1<br>No 2 *If No, go to H15*	H12
Have you ever been told by a doctor or other health worker that you have raised cholesterol?	Yes 1<br>No 2 *If No, go to H15*	H13a
Have you been told in the past 12 months?	Yes 1<br>No 2	H13b
In the past two weeks, have you taken any oral treatment (medication) for raised total cholesterol prescribed by a doctor or other health worker?	Yes 1<br>No 2	H14
Have you ever seen a traditional healer for raised cholesterol?	Yes 1<br>No 2 *If No, go to H17*	H15
Are you currently taking any herbal or traditional remedy for your raised cholesterol?	Yes 1<br>No 2	H16

**History of Cardiovascular Diseases**		
Have you ever had a heart attack or chest pain from heart disease (angina)?	Yes 1<br>No 2	H17
Are you currently taking aspirin regularly to prevent or treat heart disease?	Yes 1<br>No 2	H18
Are you currently taking statins (Lovastatin/Simvastatin/Atorvastatin or any other statin) regularly to prevent or treat heart disease?	Yes 1<br>No 2	H19
Did your mother or father have a heart attack before the age of 60?	Yes 1<br>No 2<br>Unknown 3	H20
Have you ever had a stroke (cerebrovascular accident or incident)?	Yes 1<br>No 2	H21
Are you currently taking aspirin regularly to prevent a stroke?	Yes 1<br>No 2	H22

Are you currently taking statins (Lovastatin/Simvastatin/Atorvastatin or any other statin) regularly to prevent or treat a stroke?	Yes 1<br>No 2	H23
Did your mother or father have a stroke before the age of 60?	Yes 1<br>No 2<br>Unknown 3	H24

History of cancer

Have you been treated for cancer before?	Yes	1	CA1
	No	2	
	Unknown	3	
If yes, which cancer	Free text		CA2

Quality of Life

We will now ask you some questions about your general health.w

For the following questions, please indicate which statements best describe your own health state today.

First, I would like to ask you about mobility. Would you say you have …	No problems walking about	1	Q1
	Some problems walking about	2	
	You are confined to bed	3	
Next, I would like to ask you about self-care. Would you say you have …	No problems with self-care	1	Q2
	Some problems washing or dressing yourself	2	
	You are unable to wash or dress yourself	3	
Next, I would like to ask you about your usual activities, for example work, study, housework, family or leisure activities. Would you say you have …	No problems with performing your usual activities	1	Q3
	Some problems with performing your usual activities	2	
	You are unable to perform your usual activities	3	
Next, I would like to ask you about pain or discomfort. Would you say you have …	No pain or discomfort	1	Q4
	Moderate pain or discomfort	2	
	Extreme pain or discomfort	3	
Finally, I would like to ask you about anxiety or depression. Would you say you have …	Not anxious or depressed	1	Q5
	Moderately anxious or depressed	2	
	Extremely anxious or depressed	3	

I would now like to ask you to do a different task.<br>To help you say how good or bad your health state is, I would like you to try to picture in your mind a scale that looks a bit like a thermometer. Can you do that? The best health state you can imagine is marked 100 (one hundred) at the top of the scale and the worst state you can imagine is marked 0 (zero) at the bottom.<br><br>I would now like you to tell me the point on this scale where you would put your own health state today.	Integer (limited to 0-100)	Q6

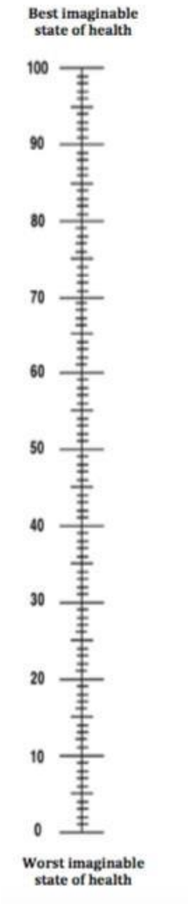

2) Behavioral measurements

Tobacco Use		
I am going to ask you some questions about tobacco use.		
**Question**	**Response**	**Code**
Do you **currently** smoke any **tobacco** products, such as cigarettes, cigars or pipes?<br>*(USE SHOWCARD)*	Yes 1<br>No 2 *If No, go to T8*	T1
Do you currently smoke tobacco products **daily**?	Yes 1<br>No 2	T2
How old were you when you **first started** smoking?	Age (years) └─┴─┘ *If Known, go to T5a/T5aw*<br>Don't know	T3
Do you remember how long ago it was?	In Years └─┴─┘ *If Known, go to T5a/T5aw*	T4a
(RECORD ONLY 1, NOT ALL 3)	OR in Months └─┴─┘ *If Known, go to T5a/T5aw*	T4b
Don't know	OR in Weeks └─┴─┘	T4c
On average, **how many** of the following products do you smoke **each day/week?**<br>*(IF LESS THAN DAILY, RECORD WEEKLY)*<br>*(RECORD FOR EACH TYPE, USE SHOWCARD)*<br>*Don't Know*	DAILY↓ WEEKLY↓	
	Manufactured cigarettes └─┴─┴─┴─┘ └─┴─┴─┴─┘	T5a/T5aw
	Hand-rolled cigarettes └─┴─┴─┴─┘ └─┴─┴─┴─┘	T5b/T5bw
	Pipes full of tobacco └─┴─┴─┴─┘ └─┴─┴─┴─┘	T5c/T5cw
	Cigars, cheroots, cigarillos └─┴─┴─┴─┘ └─┴─┴─┴─┘	T5d/T5dw
	Number of Shisha sessions └─┴─┴─┴─┘ └─┴─┴─┴─┘	T5e/T5ew
	Other └─┴─┴─┴─┘ └─┴─┴─┴─┘<br>*If Other, go to T5other, else go to T6*	T5f/T5fw
	Other (please specify): └─┴─┴─┴─┴─┴─┘	T5other/ T5otherw
During the past 12 months, have you tried to **stop smoking**?	Yes<br>No 1 2	T6
During any visit to a doctor or other health worker in the past 12 months, were you advised to quit smoking tobacco?	Yes 1<br>No 2<br>No visit during the past 12 months 3	T7
In the past, did you **ever smoke** any tobacco products?<br>*(USE SHOWCARD)*	Yes 1 *If No, go to A1*<br>No 2 *If No, go to A1*	T8
In the past, did you **ever** smoke **daily**?	Yes 1<br>No 2	T9
How old were you when you **first started** smoking?	Age (years)<br>Don't know └─┴─┘ *If Known, go to T5a/T5aw*	T10

Do you remember how long ago it was?<br><br>*Don't know*	In Years └─┴─┘ *If Known, go to T5a/T5aw*<br>OR in Months └─┴─┘ *If Known, go to T5a/T5aw*<br>OR in Weeks └─┴─┘	T11a<br>T11b<br>T11c
How old were you when you **last quit** smoking?<br><br>Do you remember how long ago it was?	Age (years) └─┴─┘ *If Known, go to T5a/T5aw*<br>Don't know<br>In Years └─┴─┘ *If Known, go to T5a/T5aw*<br>OR in Months └─┴─┘ *If Known, go to T5a/T5aw*<br>OR in Weeks └─┴─┘	T12<br><br>T13a<br>T13b<br>T13c

Alcohol Consumption

The next questions ask about the consumption of alcohol.

Question	Response	Code
Have you **ever** consumed any alcohol such as beer, wine, spirits or *[add other local examples]*?<br>*(USE SHOWCARD OR SHOW EXAMPLES)*	Yes 1<br>No 2 *If No, go to TB1*	A1
Have you consumed any alcohol within the **past 12 months**?	Yes 1 *If Yes, go to A4*<br>No 2	A2

Have you stopped drinking due to health reasons, such as a negative impact on your health or on the advice of your doctor or other health worker?	Yes 1<br>No 2	A3
During the past 12 months, **how frequently** have you had at least one standard alcoholic drink?<br><br>*(READ RESPONSES, USE SHOWCARD)*	Daily 1<br>5-6 days per week 2<br>3-4 days per week 3<br>1-2 days per week 4<br>1-3 days per month 5<br>Less than once a month 6	A4
Have you consumed any alcohol within the **past 30 days**?	Yes 1<br>No 2 *If No, go to TB1*	A5
During the past 30 days, on how many **occasions** did you have at least one standard alcoholic drink?	Number<br>Don't know └─┴─┘	A6
During the past 30 days, when you drank alcohol, how many **standard drinks on average** did you have during one drinking occasion?<br>*(USE SHOWCARD)*	Number<br>Don't know └─┴─┘	A7
During the past 30 days, what was the **largest number** of standard drinks you had on a single occasion, counting all types of alcoholic drinks together?	Largest number<br>Don't Know └─┴─┘	A8
During the past 30 days, how many times did you have **six or more** standard drinks in a single drinking occasion?	Number of times<br>Don't Know └─┴─┘	A9

During each of the **past 7 days**, how many standard drinks did you have each day?<br><br>*(USE SHOWCARD)*<br><br>*Don't Know*	Monday └─┴─┘	A10a
	Tuesday └─┴─┘	A10b
	Wednesday └─┴─┘	A10c
	Thursday └─┴─┘	A10d
	Friday └─┴─┘	A10e
	Saturday └─┴─┘	A10f
	Sunday └─┴─┘	A10g

Alcohol Consumption, continued

I have just asked you about your consumption of alcohol during the past 7 days. The questions were about alcohol in general, while the next questions refer to your consumption of homebrewed alcohol, alcohol brought over the border/from another country, any alcohol not intended for drinking or other untaxed alcohol. Please only think about these types of alcohol when answering the next questions.

Question	Response	Code
During the **past 7 days**, did you consume any **homebrewed** alcohol, any alcohol **brought over the border/from another country**, any alcohol **not intended for drinking** or other **untaxed** alcohol?<br>*[AMEND ACCORDING TO LOCAL CONTEXT]*<br>*(USE SHOWCARD)*	Yes 1<br><br>No 2 *If No, go to TB1*	A11
On average, **how many standard drinks** of the following did you consume **during the past 7 days**?<br><br>*[INSERT COUNTRY-SPECIFIC EXAMPLES]*<br>*(USE SHOWCARD)*<br><br>*Don't Know*	Homebrewed spirits, e.g. moonshine └─┴─┘	A12a
	Homebrewed beer or wine, e.g. beer, palm or fruit wine └─┴─┘	A12b
	Alcohol brought over the border/from another country └─┴─┘	A12c
	Alcohol not intended for drinking, e.g. alcohol-based medicines, perfumes, after shaves └─┴─┘	A12d
	Other untaxed alcohol in the country └─┴─┘	A12e

3) TB screening and asthma/COPD questions

TB screening questions			
Are you **currently** on TB treatment?	Yes	1	TB1
	No	2 If no, go to TB3	
If yes, have you received injections as part of this TB treatment?	Yes	1	TB2
	No	2	
	Unknown	3	
If yes, when did you start your current treatment for TB?	Date		TB2a
Have you been on TB treatment **before**?	Yes	1	TB3
	No	2 *If No, go to TB6*	
	Unknown	3 *If Unknown, go to TB6*	
If yes, how many times have you been on TB treatment?	Number of times		TB4a
Do you remember how old you were when you had your FIRST diagnosis of TB?	Yes	1 *If Yes, go to TB4c*	TB4b
	No		
Age	Years		TB4c
Do you remember the exact date or year when you completed your last TB treatment?	Exact date		TB5
	Year only		
	I don't know		
Has anyone in your homestead (or residential plot), during your lifetime, **ever** had TB disease? *(Including those who might not have started TB treatment but were told by a clinician that they had TB disease)*	Yes	1	TB6
	No	2	
	Unknown	3	

Is anybody in your homestead (or residential plot) **currently** on TB treatment?	Yes	1	TB7
	No	2	
	Unknown	3	
Do you **currently** have a cough?	Yes	1	TB8
	No	2 *If No, go to TB11*	
If yes, how long have you had the cough	Number of weeks		TB9
If yes, do you cough up sputum?	Yes	1	TB10
	No	2	
Do you have a fever?	Yes	1	TB11
	No	2	

If yes, how long have you had a fever?	Number of weeks		TB12
Do you have drenching night sweats? *(Sweating at night that soaks your clothes or beddings)*	Yes	1	TB13
	No	2 *If No, go to TB15*	
If yes, how long have you had drenching night sweats?	Number of weeks		TB14
Have you lost weight in the last 6 months? *(Explain to the participant that this is unexplained or unintentional weight loss)*	Yes	1	TB15
	No	2 *If No, go to TB171*	
If yes, how much weight have you lost in the last 6 months?	Kg		TB16
Do you have any other symptoms apart from those already mentioned? *(Prompt for TB specific symptoms [fatigue, haemoptysis, chest pain, and lymphadenitis])*	Yes	1	TB17
	No	2 *If No, go to AS1*	
If yes, which symptom	Fatigue		TB18
	Haemoptysis		
	Chest pain		
	Lymphadenitis		
If yes, duration of the symptom	Number of weeks		TB19

**Asthma / COPD questions**		
Have you ever been told by a doctor or other health worker that you have asthma or COPD?	Yes 1<br>No 2 *If No, go to AS4*	AS1
Have you been told in the past 12 months?	Yes 1<br>No 2	AS2
In the past two weeks, have you taken any medication for asthma/COPD prescribed by a doctor or other health worker?	Yes 1<br>No 2	AS3
Do you have shortness of breath after exercise or physical activity?	Yes 1<br>No 2	AS4
Do you wake up at night because of difficulties in breathing/shortness of breath?	Yes 1<br>No 2	AS5
Do you experience wheezing at night? *(Wheezing explained as a whistling sound that is made while breathing)*	Yes 1<br>No 2	AS6

4) HIV questions

**HIV questions**			
Have you ever received a test result for HIV?	Yes	1	HI1

	No	2 *If No, go to Section 5*	
	Unknown	3 *If No, go to Section 5*	
Have you ever had a positive HIV test result?	Yes	1	HI2
	No	2	
	Unknown	3	
HIV-positive			
Do you remember the exact date, or year of your first positive test result?	Exact date		HI3
	Year only		
	I don't know		
Have you ever been on ART?	Yes	1 *If Yes, go to HI5*	HI4
	No	2 *If No, go to Section 5*	
	Unknown		
Are you currently receiving ART?	Yes	1	HI5
	No	2	
	Refused to say	3	
Do you remember the exact date, or year when you first started ART did you first start ART?	Exact date		HI6
	Year only		
	I don't know		

Have you ever stopped/defaulted from ART treatment? (*Defaulted explained as not taken treatment for more than 3 months – 90 days)*	Yes	1	HI7
	No	2	
	Unknown	3	
How many times	Times		HI8
HIV-negative			
When was your last negative test result?	Date	0	HI9

5)	**Any other medical problem for which you regularly see a doctor (or traditional healer) or for which you have been prescribed a medication?**

**Medical problems**	
**Any other medical problem for which you regularly see a doctor (or traditional healer) or for which you have been prescribed a medication?**	
**Medical problem (select)**	**Date/Year started**
Hypertension	
Diabetes	
Cholesterol	
Heart attack or stroke (cardiovascular disease)	
Tuberculosis	
Asthma/COPD	
HIV	
Other	

**Concurrent medication**					
**Name medication**	**Indication (select)**	**Date/year started**	**Currently taking**	**Taking as needed**	**Not taking**
	Hypertension				
	Diabetes				
	Cholesterol				
	Heart attack or stroke (cardiovascular disease)				
	Tuberculosis				
	Asthma/COPD				
	HIV				
	Other				

6) Operational questions

**Operational questions**			
Time of last meal or non-water drink	Date and time		

For women only:			
Do you remember the exact date, or year when your last menstrual period was?	Exact date		
	Year only		
	I don’t know		
	Never		

References

1. Gunda R, Koole O, Gareta D, Olivier S, Surujdeen A, Smit T, et al. Cohort Profile: The Vukuzazi ('Wake Up and Know Yourself' in isiZulu) population science programme. Int J Epidemiol. 2022;51(3):e131-e42.
